# Features of the Retinotopic Representation in the Visual Wulst of a Laterally Eyed Bird, the Zebra Finch (*Taeniopygia guttata*)

**DOI:** 10.1371/journal.pone.0124917

**Published:** 2015-04-08

**Authors:** Neethu Michael, Siegrid Löwel, Hans-Joachim Bischof

**Affiliations:** 1 Department of Systems Neuroscience, Johann-Friedrich-Blumenbach Institut für Zoologie und Anthropologie, Universität Göttingen, Göttingen, Germany; 2 Verhaltensforschung, Universität Bielefeld, Bielefeld, Germany; 3 Göttingen Graduate School for Neurosciences, Biophysics, and Molecular Biosciences (GGNB), Göttingen, Germany; Universität Bielefeld, GERMANY

## Abstract

The visual wulst of the zebra finch comprises at least two retinotopic maps of the contralateral eye. As yet, it is not known how much of the visual field is represented in the wulst neuronal maps, how the organization of the maps is related to the retinal architecture, and how information from the ipsilateral eye is involved in the activation of the wulst. Here, we have used autofluorescent flavoprotein imaging and classical anatomical methods to investigate such characteristics of the most posterior map of the multiple retinotopic representations. We found that the visual wulst can be activated by visual stimuli from a large part of the visual field of the contralateral eye. Horizontally, the visual field representation extended from -5° beyond the beak tip up to +125° laterally. Vertically, a small strip from -10° below to about +25° above the horizon activated the visual wulst. Although retinal ganglion cells had a much higher density around the fovea and along a strip extending from the fovea towards the beak tip, these areas were not overrepresented in the wulst map. The wulst area activated from the foveal region of the ipsilateral eye, overlapped substantially with the middle of the three contralaterally activated regions in the visual wulst, and partially with the other two. Visual wulst activity evoked by stimulation of the frontal visual field was stronger with contralateral than with binocular stimulation. This confirms earlier electrophysiological studies indicating an inhibitory influence of the activation of the ipsilateral eye on wulst activity elicited by stimulating the contralateral eye. The lack of a foveal overrepresentation suggests that identification of objects may not be the primary task of the zebra finch visual wulst. Instead, this brain area may be involved in the processing of visual information necessary for spatial orientation.

## Introduction

In vertebrate brains, sensory information is often represented in an orderly manner. Our body surface is, for example, represented in the well-known homunculus in the somatosensory cortex [1]. Likewise, in the visual system, visual information reaching the retina is represented topographically in many of the target areas of the visual pathway: visual stimuli from neighboring locations in the visual field activate neighboring neurons within the visual processing area [2–4].

The origin of the visual topographic maps within the brain is the retina. Its essential organization including photoreceptors, amacrine, horizontal, bipolar and retinal ganglion cells is similar in all vertebrates [5]. It is also a common feature of all vertebrate retinae that the density of photoreceptors and retinal ganglion cells is not homogeneous [6,7]. In many cases, there is at least one, sometimes even more regions of higher density [8]. Often, a fovea is found with even more densely packed photoreceptors and retinal ganglion cells [9].

As a rule, the dimensions of neuronal retinotopic maps depend on the density of retinal ganglion cells. The foveal area of many mammals, for example cats and primates [6,10], has a high density of retinal ganglion cells and, accordingly, the part of the retinotopic map representing the fovea is larger than parts representing similarly sized areas of the retinal periphery. This is called "foveal overrepresentation" [3,10,11]. Such overrepresentation is in many cases enhanced by differential wiring of foveal and nonfoveal photoreceptors and retinal ganglion cells: Within foveal regions, single or a small number of photoreceptors project to a given ganglion cell, while in the periphery, hundreds of photoreceptors can converge onto one ganglion cell [10].

In mammals as well as in other vertebrates, it is not only one eye which is projecting to a given brain map. It has been frequently shown that information from both eyes is combined in retinotopic maps [11–13]. In many cases, binocular neurons receive information from both eyes, and the percentage of binocular neurons is related to the overlap of the visual field of both eyes: if the visual field overlap is high like in primates or cats, there is a large binocular segment in the retinotopic map [14], and a comparatively small one in laterally eyed animals with a smaller binocular overlap like mice [15].

The visual system of birds consists of three parallel projections originating in the retina [16]-tectofugal pathway, thalamofugal pathway and accessory optic system (AOS). Visual information is transported completely to the contralateral hemisphere by the optic nerve. AOS is mainly involved in the processing of optic flow induced by self-motion [17]. The tectofugal or collothalamic pathway has its first station [16] in the mesencephalic optic tectum which is organized in up to 15 layers and contains a complete retinotopic map. From there the information is transferred in a non-topographic manner to the thalamic n. rotundus which has special subdivisions[18] for color, luminance, and motion of visual stimuli [19]. The first telencephalic station is the entopallium. Entopallial lesions in pigeon have demonstrated that the tectofugal pathway is important for pattern discrimination and complex processes like concept learning or discrimination of concepts (review: [16,20]). At least for laterally eyed birds, the tectofugal pathway has been considered as the most important one because lesions of this pathway in general have massive effects in comparison with lesions of the thalamofugal or lemnothalamic pathway [16,20], which will be described in more detail below. It has to be noted, however, that there are as yet no lesion studies and no other information about its relative importance for frontally eyed birds. It is possible that in these birds the thalamofugal system, especially tuned to process binocular information (see below) may have become the more important projection, as it has been shown for the homologous geniculocortical system in mammals [21].

The aim of the present study is to investigate functional aspects of the telencephalic station of the thalamofugal pathway, the visual wulst, which receives information from the retina, relayed by several thalamic nuclei (review: [22,23][24]). In particular, we wanted to concentrate on the retinotopic representation of the visual field within this area. The experiments were performed in the zebra finch, a small songbird with laterally placed eyes and in this aspect representative of most avian tribes.

The eyes of birds are, as mentioned before, in most aspects similar to those of mammals. Special features are the so called oil droplets which contribute to the high spectral sensitivity [25,26], the pecten, a big structure protruding into the vitreous which is thought to serve for nutritional purposes [27,28], and probably a much higher variation across species in photoreceptor and retinal ganglion cell densities [9,29–31].

The visual wulst receives visual input from the retina relayed by the optic thalamic nuclei. It consists of three roughly parallel oriented layers. Visual input from the thalamus reaches the middle layer, the IHA (nucleus interstitialis hyperpallii apicale), and is transferred from this layer to the upper layer, HA (hyperpallium apicale) that sends projections back to the thalamus, and the lowest layer, HD (hyperpallium densocellulare) which is the source of intratelencephalic projections of the wulst [23]. Developmental studies indicate that these layers cannot be compared with cortical layers although the area is said to be homologue to the visual cortex [24]. Using optical imaging of intrinsic signals we have previously shown that the zebra finch visual wulst comprises several topographic maps which can be easiest detected from the IHA at a depth of around 500 μm; recording from deeper or more superficial layers revealed reduced signal-to-noise ratio [32].

Information from both eyes reaches the visual wulst in spite of the fact that, in contrast to mammals, the optic nerves cross completely to the contralateral side in birds. Pettigrew and Konishi [12] electrophysiologically recorded binocular neurons from the visual wulst of owls and described a number of neuronal features comparable with those of neurons of the mammalian visual cortex like precise retinotopic organization, direction and orientation selectivity and binocular disparity [13]. The information from the ipsilateral eye was shown to originate from a secondary recrossing of thalamic efferents to the contralateral telencephalon by means of the dorsal supraoptic decussation [22,33].

Such binocular processing within the visual wulst, however, is probably a specialty of birds with frontally placed eyes and a quite large binocular visual field. Although there is a substantial amount of recrossing fibers, the influence of the ipsilateral eye on the activation of the wulst has been shown to be marginal in birds with laterally placed eyes like the chicken and the zebra finch [34–36]. It has been speculated that the size of the wulst is related to the amount of binocularity and the size of the binocular overlap [21]. In previous electrophysiological experiments, we have found that in the zebra finch, ipsilaterally evoked activity in the visual wulst reduced activity induced by stimulating the contralateral eye [34].Birds with lateral eyes fixate objects within the environment by directing the fovea of one eye to it [37]. In such a case, a suppression of the information from the nonfixating eye may make sense because binocular processing is not advantageous [34–36].

Based on our previous report on the existence of several retinotopic maps in the zebra finch visual wulst [32], we are now aiming to describe the retinotopic visual field representations in more detail and ask the following questions: What is the extent of the visual field representation in the visual wulst? How far can the architecture of the wulst map be explained by the retinal ganglion cell distribution? Is there a foveal overrepresentation? Are there binocular interactions, i.e. does stimulation of the ipsilateral eye affect wulst activity induced by stimulating the contralateral eye? In this paper, we will focus on the most posterior map of the multiple visual field representations because it is strongly activated by visual stimuli and—in contrast to the more anterior maps—consistently visible in each imaging experiment. Instead of using optical imaging of intrinsic signals as in our previous mapping study (OIS [32]), we here used autofluorescent flavoprotein imaging (AFI [38,39]) because it is superior to the OIS method for the demonstration of retinotopic maps in the visual wulst of zebra finches as we have recently shown [40]. In addition, we performed ophthalmoscopic measurements and counted retinal ganglion cells to provide a basis for the relation between retinal architecture and wulst retinotopic maps.

## Materials and Methods

21 zebra finches of 100–110 days of age from the breeding stock of Bielefeld University were used for the imaging experiments. The retinae of additional three birds (age range 120–300 days) were used for the estimation of the ganglion cell density distribution and the measurements of retinal landmarks.

### Ethics statement

All experimental procedures were performed according to the German Law on the Protection of Animals and permitted by the local government. All imaging experiments performed in Göttingen were approved by the „Niedersächsisches Landesamt für Verbraucherschutz und Lebensmittelsicherheit”(permission numbers 84–02.04.2011.A217 and 33.9-42502-04-14/1451), all retinal ganglion cells experiments by the "Landesamt für Natur-und Verbraucherschutz" (permission AZ 87–51.04.2010.A069, May 11, 2010, LANUV, NRW).

### Optical imaging

Procedures were performed as described previously [40]. Briefly, the birds were anesthetized by injecting 0.1 ml of 20% urethane intramuscularly, and then kept under infrared light to maintain the body temperature. The birds’ heads were fixed using the stereotaxic head-holder for small birds [41], modified to hold the head in a position where the beak was oriented horizontally using ear bars and a beak holder, a modification due to the fact that the original head angle of 45° was comfortable for preparing the stereotaxic atlas, but an angle of 0° is more appropriate to mimic the natural head position of a zebra finch sitting on a perch (Bischof, unpublished). A craniotomy was performed on the left hemisphere to expose the visual wulst, leaving the dura mater intact. The exposed brain area was then covered with warm agarose (2.5% in saline) and a glass cover slip. The contralateral eye was opened by retracting and fixating the lower eyelid in an open position by Histoacryl, keeping the nictitating membrane intact. If moistening by the nictitating membrane appeared to be not sufficient, silicon oil was applied to keep the sclera in good condition.

**Fig 1:**
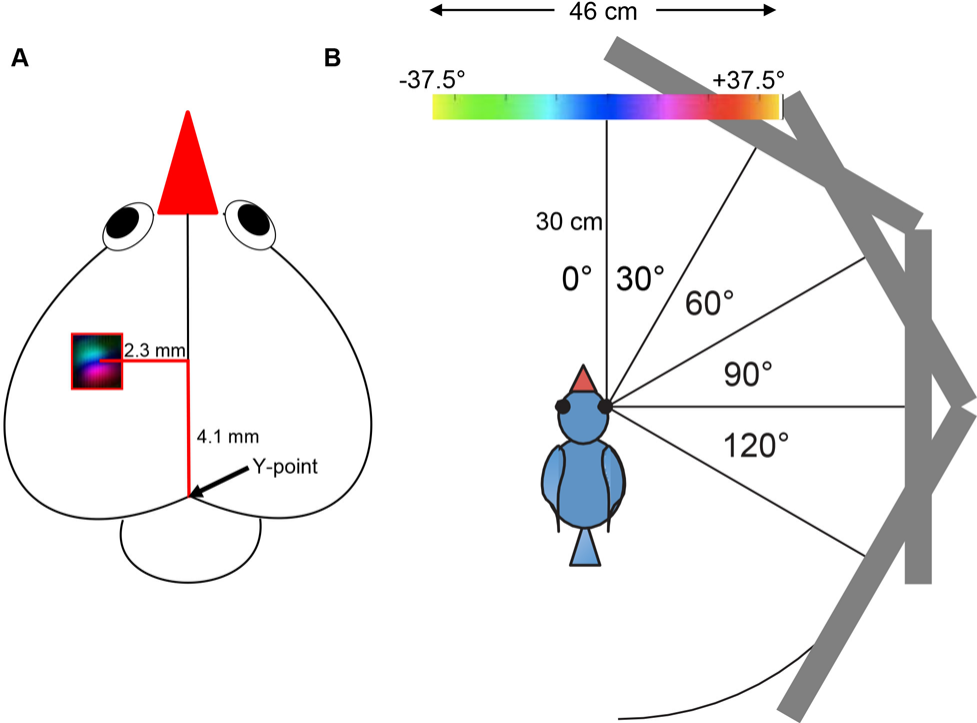
Visual stimulation conditions and location of the recorded zebra finch brain area. **A:** Positions of the stimulus monitor in the frontal and lateral visual field used in the present study. At a distance of 30 cm from the zebra finch eyes, the monitor covered 75° of visual space. In the 0° position of the monitor (0° visual field corresponded to the monitor center), the visual stimuli presented on the monitor extended 37.5° into the left (ipsilateral) and 37.5° into the right (contralateral) visual field. Likewise, in the 30° position, stimuli ranged from -7.5° to +67.5°, from +22.5° to +97.5° (60° position), from +52.5° to +127.5° (90° position) and from +82.5° to 157.5° (120° position) of the right visual field. **B:** Schematic representation of the zebra finch brain. The location of the imaged posterior map in the left visual wulst is indicated with a color-coded retinotopic map in the red rectangle; the approximate location of the center of the imaged region is given in mm distance laterally and anteriorly from the Y-point (black arrow).

Neuronal activity in the left visual wulst of zebra finches was captured using autofluorescent flavoprotein imaging [38,39]. For data acquisition and analysis we used the Fourier imaging method introduced by Kalatsky and Stryker [42]: To image sensory-driven activity, a temporally periodic stimulus was continuously presented to the bird and the brain’s response at the stimulus frequency was extracted by Fourier analysis. Optical images of visual wulst activation were obtained using a CCD camera (Dalsa 1M30) and a 130 x 55 mm lens with an aperture of 1.2 (Nikon, Tokyo, Japan), controlled by custom software. The acquired image covered 4.6x4.6 mm of the brain surface. The camera was focused below the wulst surface at a depth of 500 μm, and neuronal activity was captured using blue light (455 ±10 nm). Frames were acquired at a rate of 30 Hz, binned to 7.5 Hz and stored as 512x512 pixel images after spatial binning of the camera image. The approximate location of the center of the posterior map of the visual wulst is schematically shown in Fig 1A.

### Visual stimuli

A high refresh rate monitor (Flatron LCD 295LM, 100 Hz, 46.5×30 cm), covering 75°×50° of the visual field, was placed at various angles around the bird at a distance of 30 cm from the eyes. In the experiments examining the extent of the lateral visual field represented in the visual wulst, we used up to 5 different monitor positions in the horizontal plane (Fig 1B): 0° (in front of the bird), 30°, 60° (in a position that the fovea was directed to the center of the monitor), 90°, 120° with respect to the right (contralateral to the imaged hemisphere) eye. In case of stimulating the ipsilateral eye from the foveal direction, the monitor was positioned at 60° with respect to the left eye. For determining the frontal and binocular visual field representation, the monitor was placed at 0° with respect to the beak. Vertically, the monitor was positioned such that the horizontal line from the eye to the monitor was 7 cm above the lower rim of the monitor. In some experiments, we lowered the monitor by 15 cm. Visual stimuli consisted of vertical (azimuth) or horizontal (elevation) moving bars (4° wide), moving at a speed of 10°/sec.

### Data analysis

Activity maps were calculated from the acquired frames by Fourier analysis to extract the signal at the stimulation frequency using custom software [42]. While the phase component of the signal was used for the calculation of retinotopy, the amplitude component represents the intensity of neuronal activation, i.e. response magnitude expressed as fractional change in reflectance x10^-4^.

**Fig 2:**
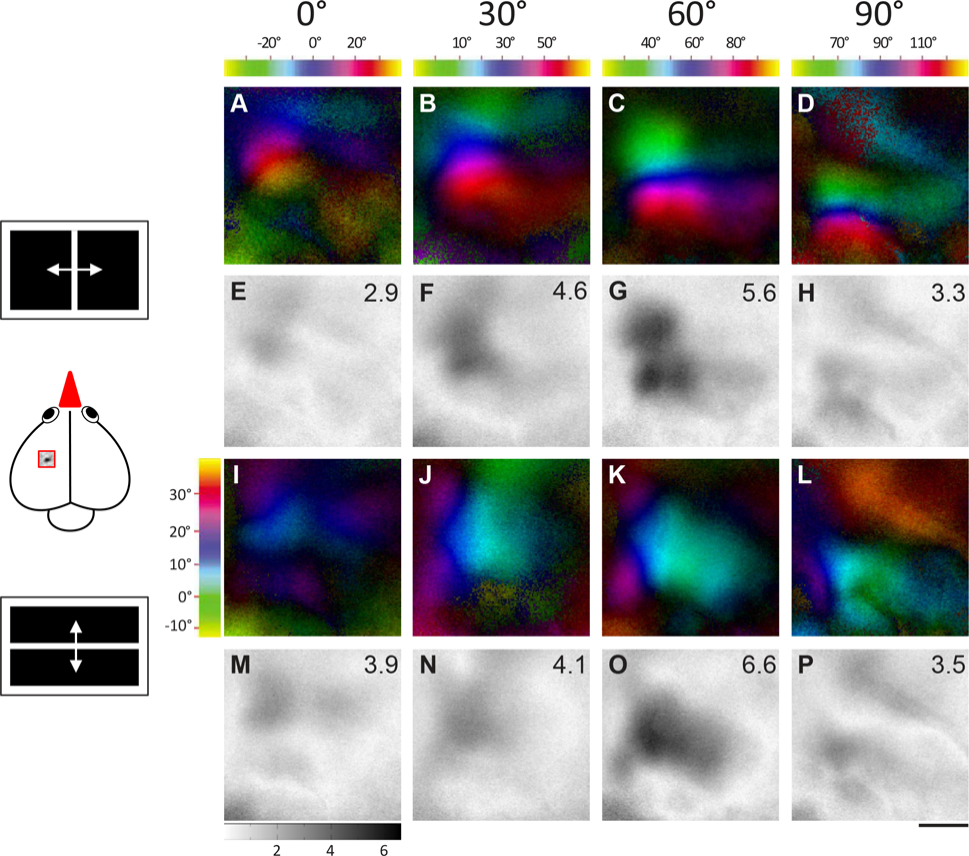
Topographic maps activated from four monitor positions demonstrating frontolateral extent of visual field representation (bird1). Activation in the left visual wulst was induced by visual stimulation of the right eye with moving vertical and horizontal bars in fronto-lateral visual-field. Colour coded polar maps of azimuth (A-D) and elevation (I-L) and grey-scale coded activity maps (E-H and M-P) recorded when the bird was visually stimulated from four different monitor positions (0°, 30°, 60°, 90°) in the fronto-lateral visual field are illustrated. The colour codes for the azimuth maps (A-D) are given above the corresponding map and for the elevation maps (I-L) on the left side of Fig 2I. Each tic on the colour scale represents 10 degrees of visual field. Wulst activation is displayed as fractional change in reflection ×10^-4^: Darker grey indicates higher wulst activation. To allow direct comparison, the grey scale at the bottom of M applies to all illustrated activity maps. The magnitude of activation is additionally shown as a number in the upper right corner of each activity map. A scheme of the visual stimuli and the approximate location of the imaged wulst area are shown on the left side of the figure. Scale bar = 500 μm.

**Fig 3:**
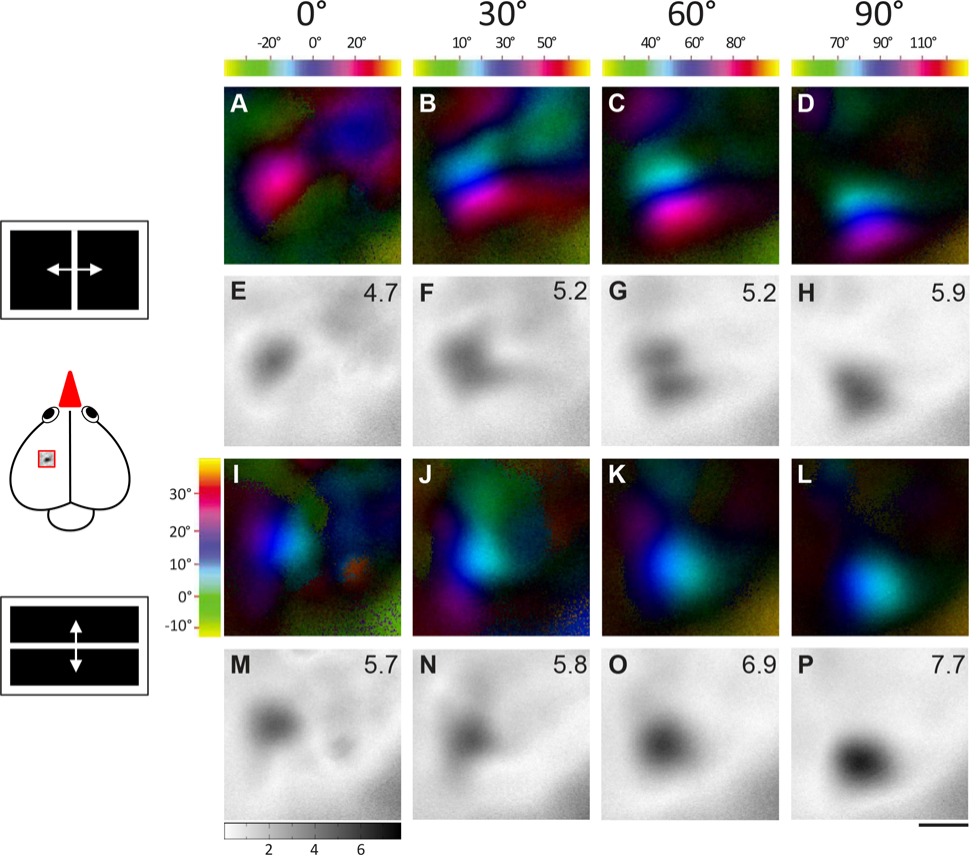
Topographic maps activated from four monitor positions demonstrating frontolateral extent of visual field representation (bird2). Data displayed as in Fig 2. **A and E** shows the azimuth polar and activity map obtained when the stimulus was presented with monitor at 0° with respect to the eye. Similarly, **B and F** shows the maps of 30° stimulation, **C and G** shows the maps of 60° stimulation and **D and H** shows the maps of 90° stimulation. In the same order **I and M**, **J and N**, **K and O**, and **L and P** shows the elevation retinotopic polar maps and the corresponding activity maps in the visual wulst when stimulated with an elevation stimulus from the different monitor positions. Scale bar = 500 μm.

**Fig 4:**
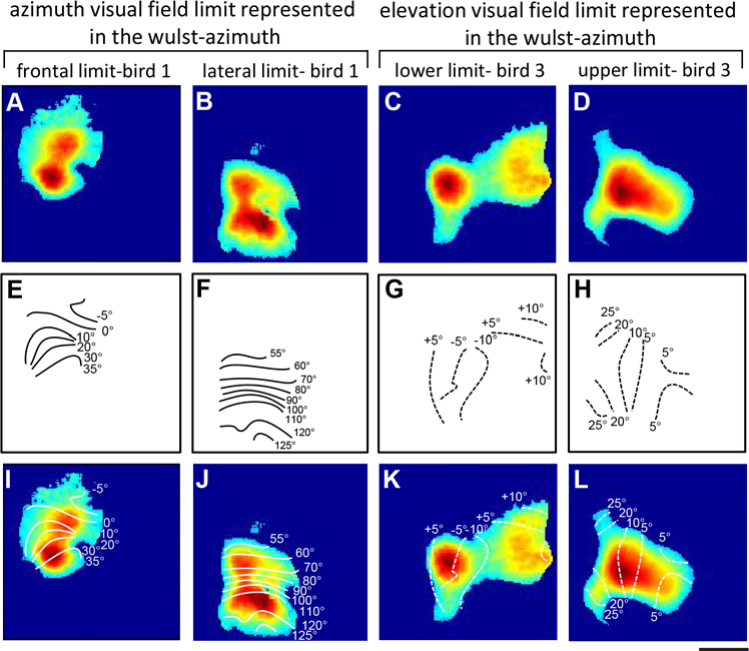
Quantitative evaluation of azimuth and elevation limits of the visual field representation. An example of the thresholded activity map of azimuth and elevation limits (A-D), its corresponding iso-azimuth and-elevation lines (E-H) and their overlay is shown (I-L). **A, E and I** shows thresholded activity map, iso-azimuth lines and their corresponding superimposition, when the monitor was positioned at 0° with respect to the eye of the bird. The iso-azimuth lines span from -5° in the ipsilateral visual field to 35° in the contralateral visual field, thus demonstrating the frontal azimuth limit of the visual field represented in the visual wulst. In the same way, **B, F and J** shows the thresholded activity map, iso-azimuth lines and their corresponding superimposition, when the monitor was positioned at 90° with respect to the eye of the bird. The iso-azimuth lines spans from 55° to 125° in the contralateral visual field, demonstrating the lateral azimuth limit of the visual field represented in the visual wulst. **C, G and K** demonstrates the lower elevation limit which is -10° below the horizon. **D, H and L** shows the upper elevation limit which is +25° above the horizon.

Retinotopic maps were colour-coded with respect to the position of the stimulus eliciting this activation. The combined information of the magnitude of neuronal activation and retinotopy is displayed in so-called polar maps. The colour codes used to display the retinotopic polar maps are shown along with each polar map. Please note that the colors in the wulst maps (Figs 2 and 3) represent different visual field positions for different monitor positions. Since blue always represents the center of the monitor, it corresponds to e.g. 0°, +30°, +60° etc. of the visual field for the azimuth maps, and to +15° for the elevation maps. To display visual field proportions in the retinotopically activated brain areas, iso-azimuth and iso-elevation lines were calculated using a Matlab routine and superimposed on the retinotopic polar maps (Fig 4). These calculations were preceded by a thresholding procedure which selected all pixels within the activity patch that showed at least 30% of the peak response, all other pixels were discarded. This procedure is illustrated in Fig 4. Fig 4A–4D gives examples of thresholded activity patches from birds 1 and 3, Fig 4E–4H shows the calculated iso-azimuth/elevation lines, and Fig 4I–4L illustrates the overlay of the thresholded activity map with the retinotopic visual field contours.

To construct the entire wulst retinotopic map of individual birds from the “partial” maps obtained from the various monitor positions, we superimposed the iso-azimuth contour lines of all maps, and—in case of overlap—displayed only the lines from the central 20° of each monitor position.

### Foveal magnification

Two parameters were used: First, we measured the size of the azimuth and elevation maps, combined the values from the two peripheral monitor positions (0° and 90°) and compared these values by t-test with the combined two”foveal” monitor positions (30° and 60°). We did this because our first impression was that the 30° and 60° segments were bigger than the peripheral ones. Second, we used a Matlab routine to evaluate the number of pixels within the activated region having the same phase value, from every 10° of the visual field starting from -5° in the ipsilateral visual field to +95° in the contralateral visual field. 30° and 60° maps were chosen for this measurement as they very well covered the area around the foveal representation and also the periphery. Since there is substantial overlap between the two monitor positions, there are more measurements/cases for each segment in the overlap region. Again, all the pixels with an amplitude lower than at least 30% of the maximum responsive pixel were discarded.

### Interaction of ipsi-and contralateral eye evoked responses

We used two parameters for the determination of evoked activity differences between bilateral and contralateral stimulation: magnitude of the neuronal activity and average amplitude of the activity. Magnitude of the neuronal activity has been explained above. In addition, we calculated the average amplitude of the wulst activation in the region of interest using ImageJ (NIH, Rasband [43]).

### Ophthalmoscopic measurement of retinal landmarks

For the inspection of retinal landmarks we used a method described in detail by Nalbach et al [44] who determined the position of retinal landmarks in the pigeon. In short, an ophthalmoscope constructed by adding additional lenses to an epi-illumination surgery microscope, was used for the inspection of the fundus of the eye. It was attached to a goniometer, which allowed the determination of azimuth and elevation of a given landmark with a precision of ± 0.1°. The values were taken relative to the optical axis of the eye which was determined by centering the corneal reflection of the epi-illumination in the pupil.

Three zebra finches were used for this study. One week before the experiment started, the birds were anaesthetized by Equithesin (2mg/kg, i.m., 0.85 g chloral hydrate, 0.21 g pentobarbital, 0.42 g MgSO_4_, 2.2 ml 100% ethanol, 8.6 ml propylene glycol, brought to a final volume of 20 ml with dH_2_O), an anesthetic found to be ideal for birds [45]. After applying a local anesthesia (xylocaine gel) to the scalp, a small incision was made on the scalp skin, and a plastic bar of 1 mm diameter and 3mm length was attached to the skull with dental cement. This bar served to fix the head of the anaesthetized bird in an adjustable clamp in a position that the observed eye was exactly located at the center of the goniometer. We measured the retinal landmarks in the left eye of all birds and also in the right eye of one of the birds. All measurements were superimposed (the right eye measurements were mirrored) by using the optical axis measurement and the beak direction.

The angular measurements could be transformed into distances on the retina by taking into account the distance from the retina to the center of the pupil (4.1mm) and using conventional trigonometric formulae. This transformation is fully correct only near the optical axis [7] but could be used to adjust the size relation between the retinal ganglion cell density map and our goniometric measurements.

### Spatial distribution of retinal ganglion cells

After finishing the ophthalmoscopic measurements, an overdose of Nembutal was injected and the zebra finches were perfused with 0.9% saline followed by 4% formaldehyde. The head of the birds was then attached to the stereotaxic head-holder. The orientation of the eye was marked by inserting an insect pin into the eye bulb in dorsoventral direction. The eye was then removed from the skull, postfixed in 10% formaldehyde, and embedded in paraffin in a way that the insect pin remained in the exact vertical position. From the paraffin blocks, 20 μm horizontal sections were cut perpendicular to the insect pin tract. By this procedure, the sections were in the same plane as the horizon which helped to interpret the results. The sections were then mounted on Superfrost slides, stained with Giemsa-solution, and cover slipped with Depex (Serva Elecrophoresis GmbH). After drying, the sections were examined using a Zeiss Axioscope under a magnification of 400x. In every fifth section, the number of retinal ganglion cells/100μm was counted along the retinal ganglion cell layer of the entire retina. The number of neurons/100x100 μm was then estimated by multiplying with five. The data were stored in a two dimensional array and processed by Sigmaplot [Systat Software Inc.] to obtain a two dimensional density map. The number of cells/mm^2^ was colour coded in steps of 2000.

No attempt was made to correct the data for tissue shrinking or counting errors [46] as we were interested in the detection of retinal areas showing enhanced ganglion cell densities, but not in the absolute numbers. We had also obtained landmarks (retina and pecten, see above) from ophthalmoscopic measurements that served to adjust the size and orientation of the obtained density map.

### Statistics

One-way repeated measures ANOVA followed by Newman-Keuls multiple comparison tests was applied to compare the magnitude of activity of the retinotopic maps from the various monitor positions and to test foveal overrepresentation. Paired t-test was used to test the difference between the activity obtained using elevation and azimuth stimuli and also the difference between the activity evoked by the stimulation of contralateral eye and binocular stimulation. Values are expressed as mean±SEM. The levels of significance were set as *: *p*<0.05; **: *p*<0.01; ***: *p*<0.001.

## Results

### Properties of the representation of the contralateral visual field

Extending our previous study about wulst retinotopic maps [32], we here focused on imaging the extent of the visual field representation in the visual wulst. To this end, we placed the stimulus monitor at five different locations from the frontal to the lateral visual field (Fig 1), and visualized wulst activation resulting from visual stimulation with moving bars (4° wide white bar). Both horizontal bars moving vertically (elevation stimulus) and vertical bars moving horizontally (azimuth stimulus) were used for determining retinotopic maps. We always recorded wulst activity from the left hemisphere and visually stimulated the right (contralateral) eye; a total of five zebra finches were used for this subproject. Figs 2 and 3 show examples of retinotopic and activity maps in two different zebra finches.

We used a vertical bar moving from left to right and from right to left (azimuth stimulus) to examine the fronto-lateral extent of the visual field represented in the visual wulst. Figs 2A, 2E, 3A and 3E illustrate the azimuth polar and activity maps of birds 1/2 when the stimulus monitor was positioned at 0°, i.e. in front of the bird. Similarly, Figs 2B, 2F, 3B, 3F, 2C, 2G, 3C, 3G, 2D, 2H, 3D and 3H represent the retinotopic and the corresponding activity maps obtained when the stimulus monitor was positioned at 30°, 60° and 90° respectively. The vertical extent of the visual field representation was examined with horizontal bars moving up and down. Figs 2I–2P and 3I–3P show the respective elevation and activity maps. The colour coding of the retinotopic maps relates to the position of the stimulus in the visual field that elicited the wulst activity and is illustrated above the maps (see materials and methods for further explanation). The magnitude of visual wulst activation is grey-scale coded with high activation represented in black; it is additionally given as a number in the upper right corner of all activity maps. Taken together, the stimulated visual field extended from -30° to +130° laterally (see scale on top of Figs 2A–2D and 3A–3D), and from -13° to +37° in the vertical direction (see scale left to Figs 2I and 3I).

In all examined cases, there was a gradual shift in the location of the activated wulst region from anterior to posterior in the zebra finch brain when the visual stimulus was presented more laterally (Figs 2 and 3). This is most easily seen by comparing the location of the “blue” region in Fig 2A–2D, corresponding to the center of the stimulus monitor, in the different azimuth maps. These polar maps confirm the presence of retinotopic order in the zebra finch visual wulst and extend previous knowledge by demonstrating that the maps extend into the periphery of the visual field.

Interestingly, there was a difference in the magnitude of the wulst activation (repeated measures ANOVA, n = 48, F = 4.132, R^2^ = 0.4525, P = 0.0021) obtained from the various stimulus monitor positions. The strongest azimuth activity map was most of the time obtained when the stimulus monitor was at 60° (3.5±0.6) with respect to the eye (e.g. Fig 2C and 2G). However, the 30° map (3.2±0.6, eg: Figs 2B, 2F, 3B and 3F) did not differ substantially from the 60° map, but the maps induced by the 0° (2.5±0.5) and 90° (2.9±0.7) monitor positions were clearly less active (eg: Fig 2A, 2E, 2D and 2H). Nevertheless, we also had two examples in which the 90° azimuth map had higher activation than the 60° maps (for eg: compare Fig 3G and 3H). The elevation maps were in general less consistent if compared with the azimuth maps. However, at least for the differences in the magnitude of activation measurements, the elevation map calculations confirmed those of the azimuth maps. Figs 2 and 3I–3P shows the elevation polar and activity maps. It was again the 60° map which showed the strongest activity in most of the cases (0°: 2.7±0.7, 30°: 3.4±0.6, 60°: 4.

Fig 4 shows two examples of how we quantified the visual field extent in the visual wulst (for details see materials and Methods). Briefly, the thresholded activity maps were superimposed with iso-azimuth and iso-elevation lines. These analyses revealed that the azimuth visual field extended from about -5° in the ipsilateral visual field (Fig 4I) to 125° in the contralateral visual field (Fig 4J), and the elevation visual field from about -10° below the horizon (Fig 4K) to +25° above the horizon (Fig 4L). These dimensions of the wulst visual field representation can also be extracted qualitatively from the colour coded polar maps illustrated in Figs 2 and 3.

**Fig 5:**
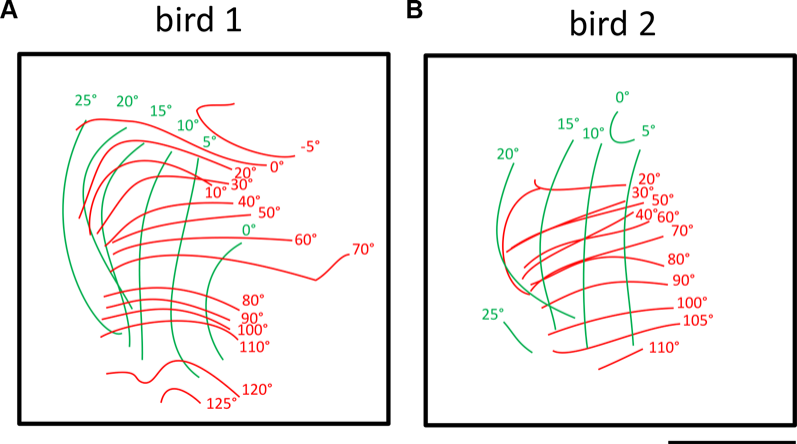
Iso-azimuth and iso-elevation lines demonstrating the extent of the visual field representation in two birds. Red complete lines denotes the iso-azimuth lines- a combined representation of maps obtained by visually stimulating with azimuth stimulus from different monitor positions. Green lines denotes iso-elevation lines- a combined representation of maps obtained by visually stimulating with an elevation stimulus from different monitor positions. **A—**bird 1, visual stimulation (both azimuth and elevation stimuli) from 0°-90°, fronto-lateral limit from -5°-+125° and vertical limit from 0°-+25°. **B—**bird 2, visual stimulation (both azimuth and elevation stimuli) from 0°-90°, fronto-lateral limit from 20°-+110° and vertical limit from 0°- +25°. Scale bar = 500 μm.

Fig 5A and 5B depict the entire representation of the visual field from birds1 and 2 (single maps illustrated in Figs 2 and 3) which were visually stimulated from -30° to 90°). In bird 1, visual stimuli from -5° to +125° medio-laterally and from 0° (horizon) to +25° above the beak induced measurable activity in the visual wulst. The visual field represented in the visual wulst of bird 2 extended from 20° to 110° laterally and from 0° to +25° above the horizon.

Although there is some variation concerning the exact extent of the visual field, our experiments show that, if one takes the extreme cases with the argument that lower values are most probably due to non-optimal experimental conditions, the horizontal extension of the visual field reaches from -5° frontally up to 125° laterally, and vertically from 10° below to +25° above the horizon. From the reconstructed maps, we determined the size of the entire posterior retinotopically organized area in the visual field as about 1.1x1 mm; the center of this representation was located 4.1 mm anterior and 2.3 mm lateral to the Y point (meeting point of the cerebellum and the two hemispheres schematically shown in Fig 1B) in the coordinate system used for the zebra finch brain atlas of Nixdorf and Bischof [47]. Although there is pronounced interindividual variability in the details of the iso-azimuth and iso-elevation contour maps, they are consistent on another feature of the retinotopic maps: as clearly visible in Fig 5, the spacing of the green iso-elevation lines is much wider than that of the red iso-azimuth lines, indicating that 10° of the visual field in the vertical plane is analyzed in about 2.5 to 3 times more neuronal space than 10° of visual field along the horizontal plane.

**Fig 6:**
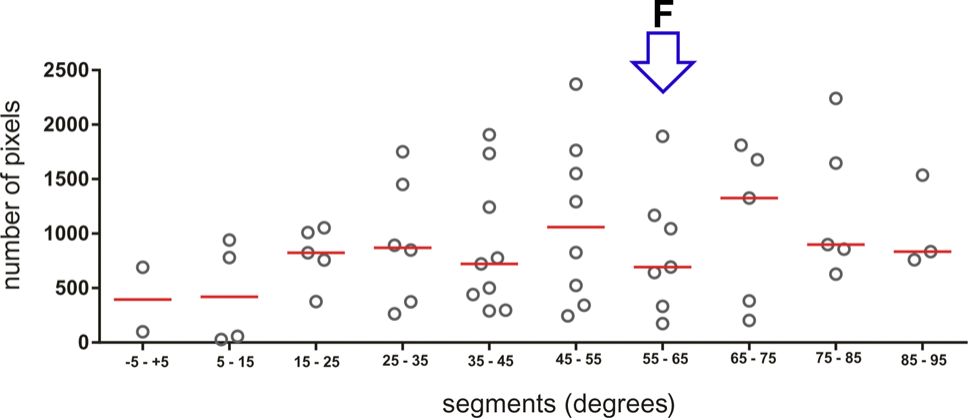
Demonstration of the lack of foveal overrepresentation. X-axis represents the various 10° wide segments of the visual field. Y axis shows the number of pixels (1 pixel = 8.89 μm) contained in the activated area. Open circles denote individual values, horizontal red lines the means of the various segments. The open blue arrow points to the segment including the foveal representation (denoted by ‘F’). There was no significant difference between the pixel numbers in the different segments. Data from five birds are illustrated.

Further comparison of the results from the different monitor positions revealed some interesting features of the intrinsic organization of the topographic representations. In mammals, as mentioned above, an overrepresentation of the foveal area has been found frequently. We therefore evaluated a potential foveal overrepresentation and also the interindividual variability of the maps between different birds by calculating the size of the areas between the azimuth lines of 10° distance along the topographic representation from -5° frontally to +95° along the horizon. Fig 6 illustrates large inter-individual differences in area sizes but no detectable foveal overrepresentation (indicated by an open blue arrow; (ANOVA, F (9, 44) = 0.7781, p = 0.6372). We also calculated the area of the activity patch induced in the visual wulst by peripheral (0°+90°) or foveal stimulation (30°+60°) (azimuth, peripheral: 0.6±0.2 mm^2^, foveal: 0.8±0.1 mm^2^; elevation, peripheral: 0.7±0.2 mm^2^, foveal: 0.9±0.2 mm^2^). Yet again there was no difference between the area sizes (repeated measures ANOVA, F (3, 6) = 0.8216, p = 0.4677).

### Retinal ganglion cell density measurements and retinal topography

**Fig 7:**
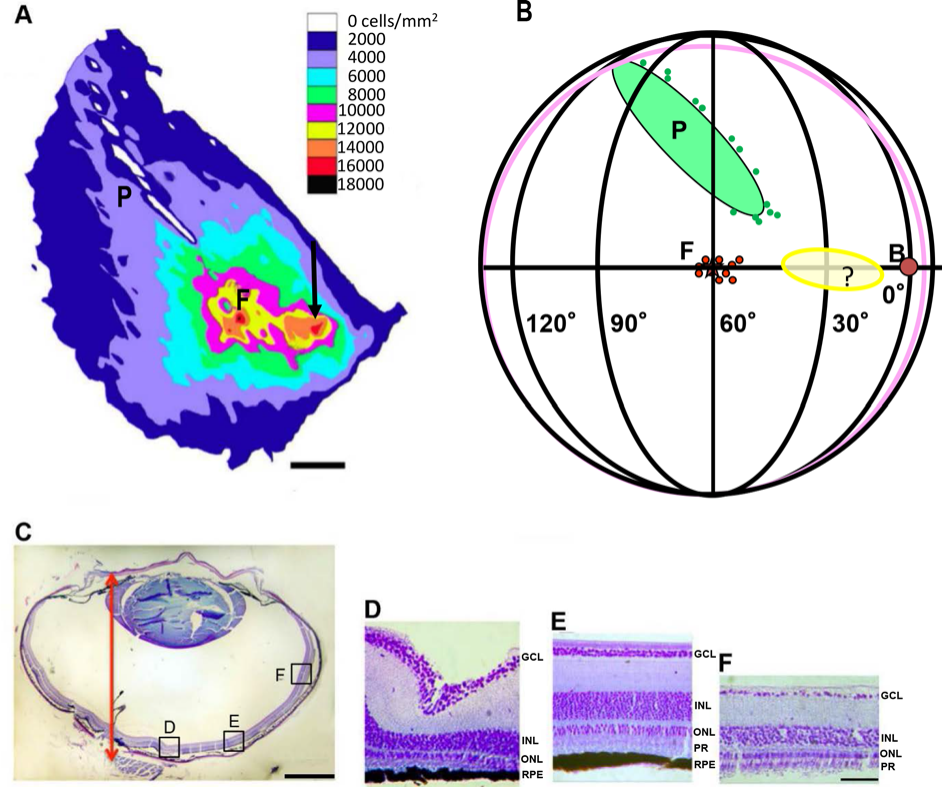
Retinal ganglion cell density measurements and landmark determination. **A—**Density distribution of retinal ganglion cells. The plot is adjusted in size and rotation to fit the landmark measurements in Fig 7B. P- pecten, F-fovea, Black arrow- area of higher density extending from the fovea into the direction of the beak. Scale bar: number of retinal ganglion cells/ mm^2^. **B-** Schematic representation of the positioning of the regions of higher ganglion densities with respect to visual field. P-pecten, F-fovea, B-beak indicated a red filled circle, yellow oval and ‘?’- area of higher density extending from the fovea into the direction of the beak. **C-** Horizontal section of the eyeball. Arrow depicts the axial length measured from the cornea to the retina. Scale bar = 1 mm. **D-F**—Sections at different locations along a section of retina (as indicated in Fig 7C), GCL-ganglion cell layer, INL-inner nuclear layer, ONL-outer nuclear layer, RPE-retinal pigmental epithelium, PR-photoreceptors. **D**-Fovea, **E-** medial between fovea and ora serrata, **F-** near the rim of the retina. Scale bar for D-F = 50 μm.

Fig 7 depicts the results of our retinal ganglion cell density measurements and the determination of the retinal landmarks by ophthalmoscopic investigation. Three zebra finches were used for this study. The calculated retinal ganglion cell density map is illustrated in Fig 7A; where in the visual field these regions of higher ganglion densities were present is illustrated schematically in Fig 7B. It shows the position of the retinal landmarks appearing as if projected through the lens onto a virtual globe representing the visual space. This means that the measurements of the fovea appear in the direction where the fovea is looking. The pecten is located ventrally in the eye, so its position in the visual field is dorsally, indicating that the pecten occludes the view of a substantial part of the dorsal visual field. Small red circles, marked by “F” indicate the foveal high density region of Fig 7A, the yellow oval corresponds to the second high density region, and the area where the pecten is positioned is shown in green. Fig 7C shows a cross-section through the whole eye, including an arrow that marks the distance used for the transformation of visual angles into distances on the retina; and Fig 7D–7F show examples of retinal cross-sections that were used for reconstructing the density map in A. In the cross sections, one can easily discern 4 layers containing cell bodies, with the ganglion cell layer (GCL) being the uppermost and relatively thin layer in each of the pictures. The density map (Fig 7A) shows how retinal ganglion cell density decreases from the fovea, which appears as a deep tapered depression in the retina (Fig 7D), to more peripheral regions (Fig 7E and 7F).

The density map illustrated in Fig 7A is a partial reconstruction of the entire retina, containing the most important areas like fovea, horizon and pecten. Our measurements reveal a tiny region of high ganglion cell density at the fovea (F) with about 18000 ganglion cells/mm^2^ (black), surrounded by regions with slightly smaller densities of 16000 cells/mm^2^ (red), and 14000 cells/mm^2^ (orange). High densities were also found at a second spot "looking" more frontally in the visual field with a maximum of 16000 cells/mm^2^ (red, marked by a white arrow). The fovea and the second dense area are connected by a strip of slightly lower cell density (12000 cells/mm^2^, yellow). This situation may be best described as a so-called visual streak [48]. At the upper part of the map there is a white stripe indicating a region free of ganglion cells, corresponding to the pecten, an eye specialization found in sauropsids, which inserts in the retina and protrudes into the vitreous.

The retinal ganglion cell density map was then slightly rotated and enlarged without distorting it to adapt it to our landmark measurements. We located the fovea (measurements, two each from the four analyzed retinae, at about 0° elevation (indicated by small filled red circles, Fig 7B), corresponding to the beak tip (when the beak is held horizontally), and 60° laterally, confirming earlier results [37]. According to our landmark measurements, the second high density area was "looking" in a direction located about 0° to 45°. Obviously, the region of higher density was not totally parallel to the horizontal plane; a slight shift to more ventral regions was observed (yellow oval, Fig 7B). The position of the pecten and its extension (measurements marked by green circles, extension by a green oval) indicated that it covers a substantial part of the dorsal visual field. We also included the measurements of the ora serrata, the peripheral rim of the retina (pink circle). However, because of the strong refraction at the periphery, these measurements are not entirely reliable.

**Fig 8:**
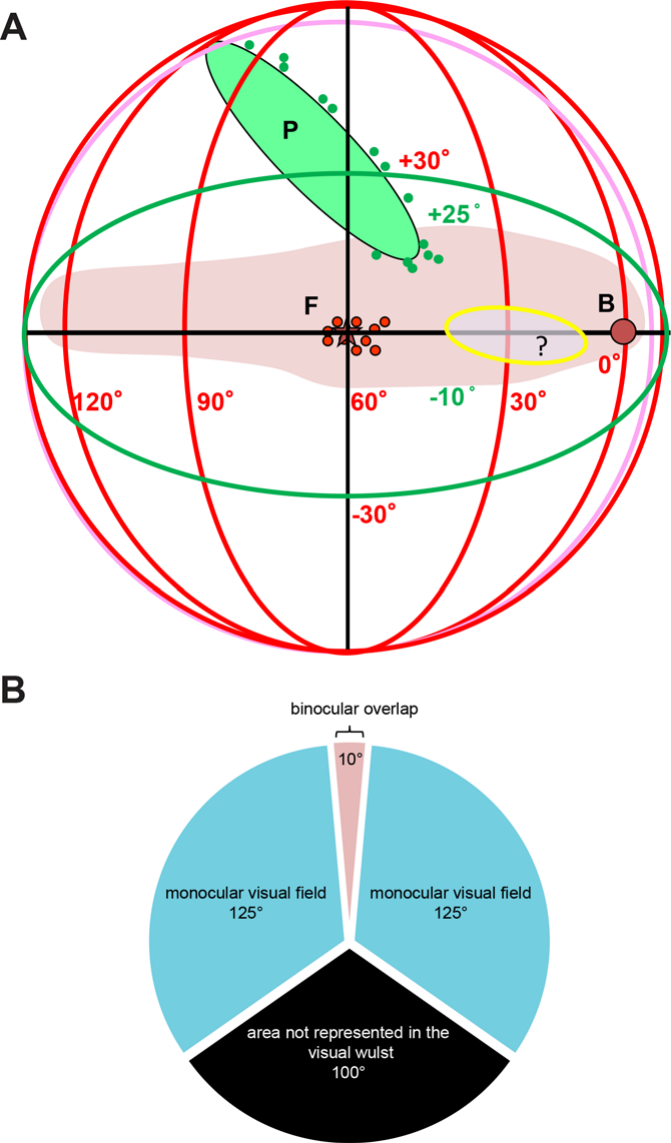
Schematic representation of the visual field extent represented in the zebra finch visual wulst. **A:** Azimuth and the elevation extent of the visual field (shaded area in pink), projected into a 3D space with lines of longitude (azimuth) and latitude (elevation). Small blue circles- points measured at the rim of the pecten, F-fovea, small red circles-measured positions of the fovea, yellow shaded oval region- area of higher density extending from the fovea into the direction of the beak. Visual field in zebra finches which is represented in the visual wulst, thus extend from around -5° frontally to +125° laterally. The visual field below the horizon up to 10° and above the horizon up to 25° is represented in the visual wulst. **B—**Demonstrates a view of the azimuthal plane along 0° latitude, which shows a binocular overlap of 10°, a lateral monocular visual field of about 125° and a caudal blind area of about 100°, which is not represented in the visual wulst.

Fig 8A summarizes our results concerning visual field extent and retinal landmarks in a schematic representation of the visual field. The pink shaded area illustrates the extent of zebra finch visual field from where visual stimuli elicited measurable activation in the visual wulst in our imaging experiments. The visual field measured about 130° in the horizontal plane, extending from around -5° in the ipsilateral visual field to about +125° in the contralateral periphery (azimuth), and about 35° in the vertical direction, extending from -10° to about +25° (elevation) between 30°-60° azimuth. It was reduced to about 15°-20° in vertical extent both frontally and more laterally. Fig 8B shows a schematic and planar bird eye’s view of the fronto-lateral visual field represented in the visual wulst. Frontally, the binocular visual field measures about 10°, the monocular left and right visual field about 130°, while a caudal region of about 100° is most likely not represented in the visual wulst.

### Ipsilateral and contralateral input to the Visual wulst

We also investigated whether the wulst gets activated by visual stimulation of the ipsilateral eye, and if so, whether the areas driven by the ipsilateral eye overlap with those activated by the contralateral eye. Given the fact that zebra finches have only a small binocular overlap, it would be difficult to comprehend if substantial regions of the wulst, which primarily represent very different parts of the visual environment would get activated by both eyes. If there is a frontal region where the visual fields of the two eyes are overlapping, afferent inputs from this visual field region could produce a binocular segment as found in mice, given that the afferents from both eyes converged on binocular neurons. We therefore examined the relation of contra- and ipsilaterally induced wulst activation in two experiments.

**Fig 9:**
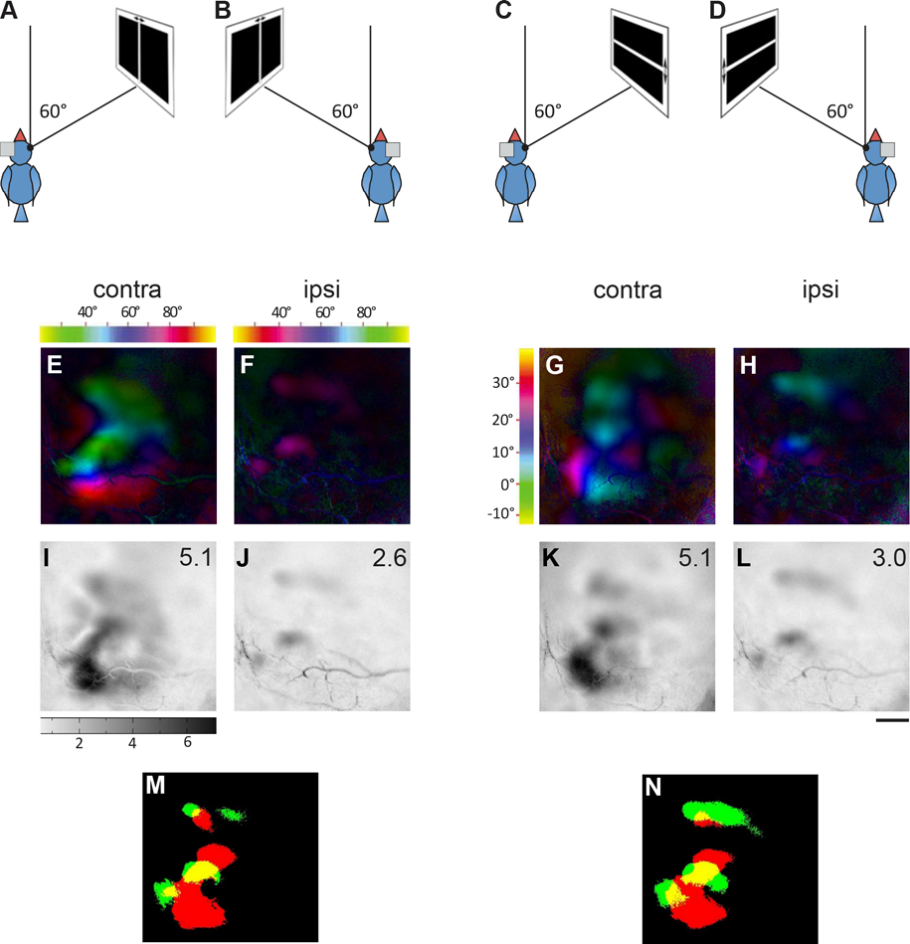
Ipsilateral foveal retinotopic map partially overlaps with contralateral one on the visual wulst. The azimuth and elevation maps obtained from the contralateral and the ipsilateral eye, when the respective eye was stimulated from an angle of 60° (foveal direction) and an overlay of the respective contralateral and ipsilateral activity maps are shown. The magnitude of activity is represented as a number in the upper right corner of the grey scale activity map (I-L). **A**—Positioning of the bird and the azimuth stimulus monitor when the contralateral eye was stimulated. The left eye was closed using a blind, the monitor was placed at 60° with respect to the right eye and the distance of the monitor from the eye was 30 cm in all cases. **B—**Positioning of the bird and the azimuth stimulus monitor when the ipsilateral eye was stimulated. The right eye was closed using a blind; the monitor was placed at 60° with respect to the left eye. **C**—Positioning of the bird and the elevation stimulus monitor when the contralateral eye was stimulated. The left eye was closed using a blind, the monitor was placed at 60° with respect to the right eye and the distance of the monitor was 30 cm in all cases. **D**—Positioning of the bird and the elevation stimulus monitor when the ipsilateral eye was stimulated. The right eye was closed using a blind; the monitor was placed at 60° with respect to the left eye. **E and I**—The azimuth polar map and grey scale activity map respectively, when the contralateral eye was stimulated with an azimuth stimulus. **F and J—**The azimuth polar map and grey scale activity map respectively, when the ipsilateral eye was stimulated. **G and K**—The elevation polar map and the grey scale activity map respectively, when the contralateral eye was stimulated with an elevation stimulus. **H and L**—The elevation polar map and the grey scale activity map respectively, when the ipsilateral eye was stimulated with an elevation stimulus. **M**—Superimposed mapping of the contralateral and the ipsilateral azimuth maps. **N—**Superimposed mapping of the contralateral and the ipsilateral elevation maps. M and N demonstrates the location of the ipsilateral map overlapping with the contralateral map. Scale bar = 500 μm.

First, the stimulus monitor was positioned at +60° (right visual field, right eye stimulation) or—60° (left visual field, left eye stimulation), and azimuth (Fig 9A and 9B) or elevation stimuli (Fig 9C and 9D) were presented. Activity was recorded in the left visual wulst. Interestingly, contralateral eye stimulation in this bird evoked multiple retinotopic maps after both azimuth and elevation stimuli (Fig 9E and 9G). While in the azimuth map (Fig 9E) separation of the different maps is difficult, there were clearly 3 separate maps in the elevation map, with the anterior two maps being inverted with respect to the posterior map, (Fig 9G). Wulst activation induced by stimulating the ipsilateral eye was weaker and more restricted in the posterior two maps (compare Fig 9I with 9J and 9K with 9L). The pink color in Fig 9F indicates that ipsilateral activation was induced by stimuli at around 50° to 40° azimuth in the left visual field. The activity patches induced by contralateral azimuth stimuli did not differ from those induced by elevation stimuli. The same was true for activation induced by azimuth and elevation stimuli via the ipsilateral eye. Fig 9M and 9N illustrates the superimposition of thresholded contra- (red) and ipsilaterally (green) evoked activity regions. It can easily be seen that there are separate areas activated by the contra- and ipsilateral eye, but there was also substantial overlap in the middle and the most posterior activated regions, visible as yellow patches in Fig 9M and 9N.

**Fig 10:**
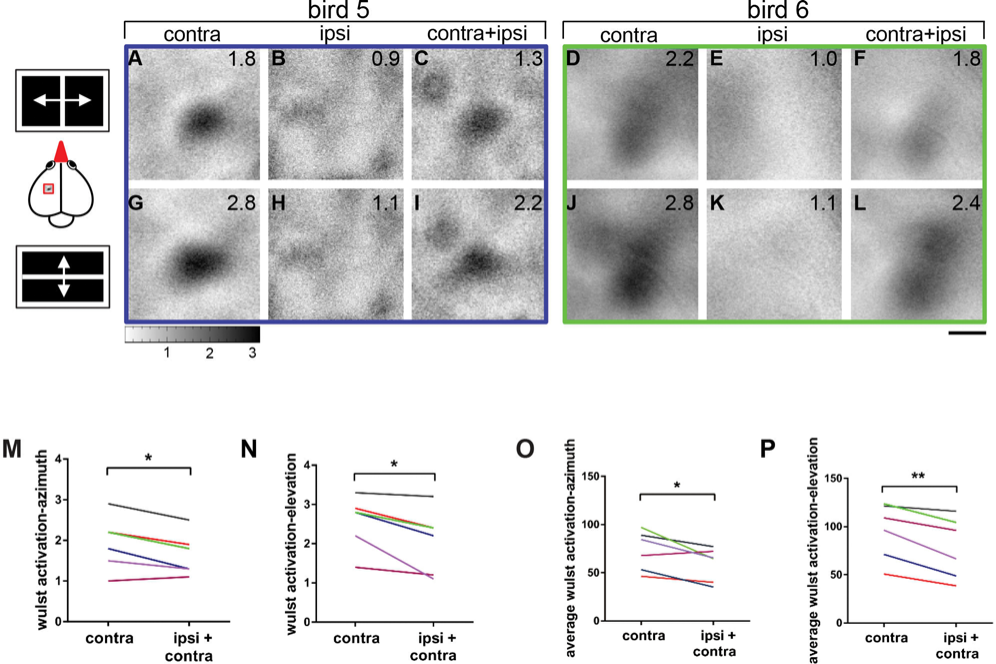
Simultaneous input from both eyes reduces the contralateral eye evoked activity in the visual wulst. Examples from two birds for the azimuth and elevation maps obtained by visually stimulating contralateral and the ipsilateral eye, when stimulated from an angle of 0° (frontal direction) and also the maps obtained when both eyes were open are shown. The magnitude of activity is represented as a number in the upper right corner of the grey scale activity map. The schematic representation of the stimulus used is shown on the left side of the corresponding maps and the distance of the middle of the monitor was always maintained as 30 cm. **A-C** and **D-F**—The azimuth activity map obtained respectively when the contralateral, ipsilateral and both eyes simultaneously, were stimulated with an azimuth stimulus at 0° with respect to the beak, from two birds. Ipsilaterally evoked activity was always lower when compared to that of the contralateral eye (compare A and B and D and E). Simultaneous input from both eyes reduces the contralaterally evoked activity in the visual wulst (compare A and C and D and F). **G-I** and **J-L**—The elevation activity map obtained respectively when the contralateral, ipsilateral and both eyes simultaneously, were stimulated with an elevation stimulus at 0° with respect to the beak. Similar to the azimuth result, ipsilateral eye showed very feebly evoked activity in the visual wulst when compared to the contralateral eye (compare G and H, J and K). Simultaneous input from both eyes reduces the contralaterally evoked activity in the visual wulst (compare G and I, J and L). **M—**Difference in the magnitude of wulst activation when the contralateral eye was stimulated alone and both ipsilateral and contralateral eyes were simultaneously stimulated with an azimuth stimulus. **N**—Difference in the magnitude of wulst activation when the contralateral eye was stimulated alone and both ipsilateral and contralateral eyes were simultaneously stimulated with an elevation stimulus. In both azimuth and elevation stimulation, there was a significant difference in the activity evoked by contralateral eye stimulation and simultaneous stimulation of both eyes. **O—**Difference in the average wulst activation, when the contralateral eye was stimulated alone and both ipsilateral and contralateral eyes were simultaneously stimulated with an azimuth stimulus. **P—**Difference in the average wulst activation, when the contralateral eye was stimulated alone and both ipsilateral and contralateral eyes were simultaneously stimulated with an elevation stimulus. The blue and green rectangular boxes around the maps indicate the examples chosen from the graphs shown below. Scale bar = 500 μm.

After having shown that visual stimuli positioned about 40 to50° laterally and -5-+25° vertically to the ipsilateral eye can activate the visual wulst, we next tested whether there is also a binocular wulst region activated from the frontal visual field, as present in mammals, and what effect binocular visual stimulation had on wulst activity. Six zebra finches were used for this experiment. The stimulus monitor was centered at 0°, in front of the beak. Both horizontal and vertical moving bars induced activity in approximately the same wulst location of the posterior map (compare Fig 10A with 10G, and 10D with 10J). Ipsilateral eye stimulation elicited only weak wulst activation in bird 5 (Fig 8B and 8H) and no discernable activation in bird 6 (Fig 10E and 10K). Interestingly, however, in both birds, binocular visual stimulation induced a wulst activation that was reduced compared to stimulation with the contralateral eye only (compare Fig 10A/10G with 10C/10I, and 10D/10J with 10F/10L). The average wulst activation after contralateral stimulation with an azimuth stimulus decreased from 1.98±0.27 (contra only) to 1.73±0.23 (contra+ipsi) (p<0.05, t-test, n = 6, Fig 10M) with binocular stimulation, and from 2.54±0.35 (contra only) to 2.24±0.39 (contra+ipsi) (p<0.05, t-test, n = 6, Fig 10N) for elevation. Thus, the ipsilateral eye exerted a measurable inhibitory influence on wulst activity when both eyes were stimulated together in the frontal visual field. As proposed for the whole wulst area based on previous electrophysiological results [34], contralaterally evoked activity was thus reduced if there was an additional input from the ipsilateral eye. As an additional parameter to test the relation between the contralaterally evoked activity and activity evoked by simultaneous stimulation of both eyes, we calculated the average wulst activity in a region including the activity patches evoked by both azimuth and elevation stimuli using the ImageJ software (see materials and methods). Similar to the activity measurements described above, there was a significant difference between the two conditions (azimuth: contralateral: 73±8.4, contra+ipsi: 59.1±7.1, p<0.05, t-test, n = 6, Fig 10O; elevation: contralateral: 95.4±11.9, contra+ipsi: 78.4±12.9; p<0.01, t-test, n = 6, Fig 10P).

## Discussion

Our imaging experiments indicate that the posterior retinotopic map of the visual wulst, represents only part of the zebra finch visual field (Fig 8), extending horizontally from -5° to about +125° on the caudal border, its vertical extension spans from about -10° (below the horizon) to about +25°. Thus, visual stimuli from only a small strip along the horizon can visually stimulate the imaged wulst region. The overlap of the representations of the left and the right eye is around 10°, and our experiments with contra- and ipsilateral stimulation indicate that there could be a binocular segment characterized by binocular neurons as it has been demonstrated in mammals. While our cell density measurements show an increased number of retinal ganglion cells at around +60° laterally and along the horizon, our reconstructions of iso-elevation and iso-azimuth lines do not give clear indications of a foveal overrepresentation in the wulst. However, the iso-elevation lines are more widely spaced than the iso-azimuth lines throughout the entire wulst visual field representation. This means that a circular object within the visual field should activate an oval region in the wulst, with the longer axis aligned to the vertical axis. In the binocular visual field, information from the ipsilateral eye seems to inhibit that of the contralateral eye; whether this is also true for lateral parts cannot be derived from our present results. Visual stimuli in the lateral parts of the visual field around the fovea, evoked small activity patches from the ipsilateral eye, partially overlapping with the contralaterally evoked activity.

### Extent of the wulst visual field representation

Our experiments demonstrated that the wulst visual field representation reached frontally slightly into the ipsilateral visual field (-5°) and was laterally limited at +125°. Vertically, it reached from +25° above the horizon down to -10° below the horizon. By using eye aperture studies, Bischof [37] had determined the visual field of the zebra finch to extend from -12.5° to +150° laterally. The difference in visual field extent to the present imaging results may have several reasons. First, unanaesthetized birds which are able to move their eyes into the direction of measurement were used in the previous study, while the birds employed for imaging in the present study were anaesthetized by urethane which has been shown to cause negligible eye movements [49]. We have confirmed the latter by recording eye movements during imaging experiments using an infrared camera (data not shown). According to Voss [50], awake zebra finches can move their eyes up to ±16.5° in any direction, which may be subtracted from the Bischof [37] results to compare the data with the present data from birds without eye movements; by doing this, the results of both studies appear rather similar. Second, one cannot be sure whether measurements using the eye aperture give real information about the area which can be processed by the eye. However, our retinal measurements indicate that the ora serrata (pink line in Figs 7B and 8A), the line which separates the receptive part of the retina from the non-receptive, is at about -15° at the frontal limit of the visual field and at about 150° at the caudal limit, just confirming the earlier measurements provided by Bischof [37]. Next, the activity evoked by stimuli at the rim of the visual field may have been smaller than that evoked by the central parts and too small to be detected by our method and finally, it is possible that the stimuli used might not have activated enough wulst neurons. Keary et al. [32] reported earlier that only stimuli presented at an elevation between 17° and 42° (a total elevation extent of 25°) could elicit activity in the visual wulst. Though the upper and lower limits of the elevation visual field is somewhat different from what we report here, the overall extent of the visual field around the foveal region is similar to the present results. The discrepancy in the upper and lower limits can be explained because the center of the stimulus monitor was taken as 0° in [12], while 0° elevation corresponds to the horizon in our study.

According to our new imaging experiments, the vertical extent of the wulst visual field representation is rather small and corresponds to just a narrow strip along the horizon (-10 to +25°). This cannot be explained by measurement differences because the upper and lower limits of the avian visual field are much higher and lower than the limits measured here [51]. Our experiments therefore unequivocally show that not the entire visual field is represented in the visual wulst map, a result which is in accordance with findings in the pigeon examining the projection from the retina to the thalamus [52], and also with lesion studies [53] demonstrating that lesion of the thalamic nuclei affect lateral, but not frontal vision.

One has to consider here that the visual wulst map is not the only brain region representing the visual field of the birds. It has been shown that the tectal map most likely represents the entire visual field [20,54–56]. Similar differences between representations have also been observed in mammals. In the cat, the representation is largest in area 17 (V1) and smallest in Brodman area 21a. There are also areas with representations elongated along the horizontal meridian (e.g. VLS-ventral lateral suprasylvian area or PLLS—posterolateral lateral suprasylvian area, review: [14]). The exact extent of the visual field representation of a particular area may thus be related to the special processing tasks of this area. What may be the function of a visual representation restricted to a rather narrow stripe along the horizon, as observed here for the visual wulst? It has been speculated that the existence of a visual streak—as we have found it in the retina and which contributes to the projections to the visual wulst map—could enhance the spatial resolution of this area, which may be advantageous for the perception of moving objects at the height of the horizon [8]. It could also be speculated that this elongated area along the horizon has to do with the feeding behaviour of zebra finches. As reported by Bischof [37], zebra finches fixate a grain that they want to eat by the fovea, then make a turn of the beak towards the grain which most probably leads to an activation of the wulst area along the strip of higher retinal ganglion cell density and correspondingly the corridor detected by the wulst map, and then perform a ballistic movement with eyes closed towards the grain (see also below). A similar idea was raised by Moroney and Pettigrew [57] for kingfishers, which fixate the prey with a central fovea and then make a head movement to transfer the image to the temporal fovea looking frontally, before starting to catch it. In both cases, a higher density of neurons may facilitate such a transfer. Already in 1958, Duijm proposed that the visual streak might be used "in the accurate establishment of the position of the eyes and therefore for compensatory eye movements and spatial orientation" [58]. Mathews also speculated about a role in navigation, serving as a baseline to which other objects in the visual field can be referred [59].

The functions which have been attributed to the visual streak could also be the basis for an explanation of the visual wulst function if it was only the streak which is projecting to the visual wulst. This, however, is not the case. The visual field represented in the wulst map extends more dorsally than the streak and also reaches into the field lateral of the fovea. Thus, the enhanced density of ganglion cells along the horizon should, if the entire population of neurons was projecting to the visual wulst, result in a stronger representation within the retinotopic map. Especially, the fovea should show some overrepresentation. However, our wulst imaging experiments do not show this. According to our results, visual stimuli at 60° (foveal) as well as at 30° (roughly the second higher density region) indeed resulted in higher magnitudes of wulst activation which in turn may indicate that neurons within this area have a higher preference for the stimuli used in our experiments. But we did not observe indications of a spatial overrepresentation of foveal regions as it is present in the mammalian visual cortex. In macaque monkeys, the central 10% of the visual field are for example represented in 90% of the V1 area, in cats it is 50% [60]. Such an overrepresentation was also found in other visual areas of the cat [61], but there are also examples of an underrepresentation [62]. In the mouse, no overrepresentation of any part of the visual field were detected; however, in this case this may be due to the lack of a retinal fovea in this species [42].

The findings in cats (over- and underrepresentation) indicate that an increased density of retinal ganglion cells does not necessarily cause overrepresentation of this region in all brain areas involved in visual scene analyses. Most likely, each area is “just” accessing the information which is necessary to perform a certain task. V1 of mammals is thought to be involved in stimulus identification, and such a task needs a high spatial resolution as provided by foveal overrepresentation. Following this reasoning, the lack of a distinct foveal overrepresentation in the zebra finch visual wulst may indicate that foveal processing and stimulus identification are not the main tasks of the visual wulst.

Given some preference for the fronto-foveal region, it rather appears that the wulst processes information from the visual field just along the horizon, and is not especially equipped for object identification which would most probably make use of the higher ganglion cell density within the fovea. We therefore speculate that approaching objects might be processed by the visual wulst. This is a testable hypothesis, and it fits well to a concept which has been raised some time ago by Watanabe and Bischof [63]. On the basis of lesion experiments, it was claimed that the visual wulst may process the "where" of an object, while the second visual pathway and its telencephalic target, the entopallium, is processing the "what", i.e. the identification of an object. Consistent with this idea, the optic tectum that projects to the entopallium comprises a foveal overrepresentation, in contrast to the visual wulst. Watanabe et al. [63] have shown that the visual wulst is feeding into the hippocampus and is therefore a candidate for mediating visual information necessary for orientation in space. This hypothesis is also supported by studies demonstrating that the visual wulst participates in sun compass orientation of pigeons [64], and in earth magnetic field orientation (mediated by the thalamofugal visual system) of both European robins [65], zebra finches [66] and garden warblers [67–69].

The vertical limitation of the wulst visual field to a rather narrow stripe along the horizon is, however, not explained by this scenario. The same ideas which have been discussed above for an explanation of a visual streak may also apply here as well as the lesion based results concerning the visual wulst function [63]. Both explanations, the idea of a role of the wulst for feeding behaviour and the more global one as a mediator of visual information for spatial orientation, are not mutually exclusive. We prefer the interpretation of a more global role of the visual wulst compared to just a feeding controller.

An interesting feature of the wulst posterior retinotopic maps is the higher magnification in the elevation compared to the azimuth direction: Ten degrees of visual space in the elevation direction were represented in a two to three times longer distance on the wulst compared to the azimuth direction. Such anisotropy of maps has also been found in a variety of mammals [70–72], but in contrast to our results, the azimuth magnification factor was in all cases bigger than that of the elevation. Ng et al., however, [73] reported that the activation of the visual wulst of pigeons is stronger if stimulated with vertically oriented stripes moving horizontally and explain it by an overrepresentation of vertical contours, which would fit to our results. Although all authors dealing with this question assume that overrepresentation of certain features are due to reflect natural visual scene statistics [70–73] there is as yet no conclusive idea explaining the potential difference between birds and mammals.

### Interaction of both eyes?

As mentioned above, the topographic maps within the mammalian visual cortex are partly receiving input from both eyes: within the so-called binocular segment, neurons respond to visual activation of both eyes. The extent of the binocular segment depends on the eye position of the animal. Mammals with laterally placed eyes and a small binocular overlap, i.e. a small frontal region where the visual fields of both eyes overlap, have a small binocular segment in their visual cortex compared with animals with more frontally positioned eyes with a larger binocular overlap. The basis of binocularity is a partial decussation of the optic nerves from both the eyes at the optic chiasm: in animals with a binocular zone, only ganglion cell axons from the nasal part of the retina cross to the contralateral hemisphere, whereas axons from temporal retina do not cross, but project ipsilaterally. The bigger the overlap of the visual field, the bigger is the binocular segment within the visual cortex. In contrast to mammals, in birds, the optic nerves cross completely to the contralateral side. Hence, ipsilateral stimulus processing and binocular interaction as shown in the visual cortex can be achieved only by a secondary re-crossing of fibers to the ipsilateral brain hemisphere. This actually occurs at both visual projections, the tectofugal [33,74] and the thalamofugal pathway [22,33]. As mentioned above, binocular interactions have been shown in owls which have quite frontally placed eyes and a substantial overlap of the two eye’s visual fields. In contrast, there is as yet no evidence for binocular processing in zebra finches. However, there are differences between results from anatomical studies [33] showing a substantial projection originating in the ipsilateral eye, and electrophysiological studies [34] indicating that this projection does not have much impact on wulst activation. By inactivating the contralateral wulst during recordings of ipsilateral stimulus responses, Bredenkötter and Bischof [34] found indications of a mutual inhibition between the two hemispheres resulting in a suppression of the ipsilateral input that increased during development [35]. They also demonstrated that inactivation of the contralateral eye did not enhance the wulst activation on ipsilateral stimulation. It is thus plausible to assume that the suppression of the ipsilateral input occurs between both hemispheres and is not a pure bottom-up effect. There is no direct connection between the wulst areas of both hemispheres. Therefore, the interaction may probably be accomplished via back projections from the wulst to the thalamic nuclei which also receive retinal input and project bilaterally to the visual wulst [22,75,76]. Mutual inhibition [77], of both hemispheres as proposed here is comparable to interocular inhibitory effects in cats [78]. The idea of a suppression of the less active eye to favour the more important information [50,77,79,80] is thus not restricted to the avian visual system.

Our results concerning the role of the ipsilateral input partly support the idea of such a suppression, but only for the "binocular segment" as discussed below. When stimuli were presented in the foveal region (60°), we observed a strong response from the contralateral eye—as expected. In contrast, stimulation of the ipsilateral eye elicited a much weaker and spatially much more restricted wulst activation (Fig 9). The fact that the area of ipsilateral activation was not totally overlapping with the area of activation evoked by the contralateral stimulus, casts doubts on the whole idea of a global mutual suppression of the contralaterally evoked activity by the ipsilateral input [34]. Obviously, the suppression is more specific. However, we are not yet able to provide an idea about how this specificity may be expressed. Due to experimental restrictions, we were not able to examine the direct interaction of simultaneous ipsi- and contralateral eye stimulation with laterally placed stimuli.

As mentioned above, the idea of a repression of activation of one eye by that of the other applies for our experiments with frontal stimulation. In these experiments, wulst activity was considerably reduced (Fig 10A–10L) when both eyes were stimulated simultaneously, compared to trials in which only the contralateral eye was stimulated. Ipsilateral eye stimulation elicited only very weak wulst activation in some of the birds preventing a detailed analyses of map overlap.

Schmidt and Bischof [81] have demonstrated lower responses with bilateral stimulation compared with pure contralateral stimuli for the n. rotundus and the entopallium of the zebra finch: about 30% of the neurons recorded in the two areas showed this response pattern, in 40% of the cases, the ipsilateral stimulus enhanced the contralateral one, and there was no effect in the rest of the cases. Thus, the inhibitory interaction of both eyes seems to be a phenomenon observed in the entire visual system of zebra finches and is most probably not restricted to a potential binocular segment. Howarth et al. [82] recently described "binocular" neurons in mice that did not respond to ipsilateral stimulation, but, in contrast to our case, ipsilateral stimuli enhanced responses to the contralateral eye if given simultaneously. Neurons with strongly reduced responses upon binocular stimulation were—however—also reported. Scholl et al. [15] convincingly showed that neurons within the binocular segment are detecting disparities between the left and the right eye, and neuronal responses were either enhanced or diminished (compared to monocular stimulation with the same moving grating) by experimental variation of the disparities. Given the fact that such disparity neurons have been demonstrated in owls [13], it might be possible that the "binocular segment” in the zebra finch wulst also contains disparity neurons, although—according to behavioural studies—laterally eyed birds do not use the frontal eye field for distance estimation [83]. Rather, as Voss and Bischof [50] have claimed, the binocular overlap may serve to keep the internal representation of the visual scene intact because, if there were no overlap, a frontal "gap" in the visual field might arise with extensive lateral eye movements. Further experiments, showing the response characteristics of single neurons within the representation of the frontal visual field of laterally eyed birds, will be necessary to clarify the role of this part of the zebra finch visual wulst.
